# Expiratory high-frequency percussive ventilation: a novel concept for improving gas exchange

**DOI:** 10.1186/s12931-022-02215-2

**Published:** 2022-10-15

**Authors:** Ferenc Peták, Gergely H. Fodor, Álmos Schranc, Roberta Südy, Ádám L. Balogh, Barna Babik, André Dos Santos Rocha, Sam Bayat, Davide Bizzotto, Raffaele L. Dellacà, Walid Habre

**Affiliations:** 1 Department of Medical Physics and Informatics, University of Szeged, 9, Korányi fasor, Szeged, 6720 Hungary; 2 Unit for Anaesthesiological Investigations, Department of Acute Medicine, University of Geneva, Geneva, Switzerland; 3 Department of Anaesthesiology and Intensive Therapy, University of Szeged, Szeged, Hungary; 4 Univ. Grenoble Alpes, Inserm UA07 STROBE Laboratory & Department of Pneumology and Clinical Physiology, Grenoble University Hospital, Grenoble, France; 5 Dipartimento Di Elettronica, Informazione E Bioingegneria, Politecnico di Milano, Milan, Italy; 6 Paediatric Anaesthesia Unit, Department of Acute Medicine, University Hospitals of Geneva, Geneva, Switzerland

**Keywords:** Gas exchange, Lung ventilation, Blood gas, Capnography, Alveolar recruitment

## Abstract

**Background:**

Although high-frequency percussive ventilation (HFPV) improves gas exchange, concerns remain about tissue overdistension caused by the oscillations and consequent lung damage. We compared a modified percussive ventilation modality created by superimposing high-frequency oscillations to the conventional ventilation waveform during expiration only (eHFPV) with conventional mechanical ventilation (CMV) and standard HFPV.

**Methods:**

Hypoxia and hypercapnia were induced by decreasing the frequency of CMV in New Zealand White rabbits (n = 10). Following steady-state CMV periods, percussive modalities with oscillations randomly introduced to the entire breathing cycle (HFPV) or to the expiratory phase alone (eHFPV) with varying amplitudes (2 or 4 cmH_2_O) and frequencies were used (5 or 10 Hz). The arterial partial pressures of oxygen (PaO_2_) and carbon dioxide (PaCO_2_) were determined. Volumetric capnography was used to evaluate the ventilation dead space fraction, phase 2 slope, and minute elimination of CO_2_. Respiratory mechanics were characterized by forced oscillations.

**Results:**

The use of eHFPV with 5 Hz superimposed oscillation frequency and an amplitude of 4 cmH_2_O enhanced gas exchange similar to those observed after HFPV. These improvements in PaO_2_ (47.3 ± 5.5 vs. 58.6 ± 7.2 mmHg) and PaCO_2_ (54.7 ± 2.3 vs. 50.1 ± 2.9 mmHg) were associated with lower ventilation dead space and capnogram phase 2 slope, as well as enhanced minute CO_2_ elimination without altering respiratory mechanics.

**Conclusions:**

These findings demonstrated improved gas exchange using eHFPV as a novel mechanical ventilation modality that combines the benefits of conventional and small-amplitude high-frequency oscillatory ventilation, owing to improved longitudinal gas transport rather than increased lung surface area available for gas exchange.

**Supplementary Information:**

The online version contains supplementary material available at 10.1186/s12931-022-02215-2.

## Background

Under general anesthesia and in critically ill patients, intermittent positive pressure ventilation is applied regularly to guarantee oxygen supply and promote carbon dioxide elimination. While maintaining physiologic lung ventilation in patients with healthy lungs is feasible, various pulmonary disorders pose challenges to health care professionals to guarantee adequate gas exchange during invasive ventilatory support. Particularly, diseases with decreased functional residual capacity and/or decreased lung compliance observed in obese patients [1, 2] or in those with acute respiratory distress syndrome (ARDS) [3, 4] require continuous adjustment of the ventilation strategy to maintain appropriate gas exchange. Furthermore, gas exchange abnormalities during mechanical ventilation have been seen in clinical situations associated with significant lung restriction and impaired gas exchange, such as during laparoscopic surgery in patients with capnoperitoneum [5, 6] or bariatric surgery [7].

Despite significant advancements in respiratory management, these increasingly common medical disorders continue to place a strain on healthcare systems by increasing the incidence of perioperative respiratory complications [8]. As a result, there is an urgent need to enhance ventilation support modalities without posing additional stress and strain on lung tissues. To enhance gas exchange, high-frequency percussive ventilation (HFPV) based on a combination of conventional and high-frequency oscillatory ventilation has been proposed [9] and applied subsequently in experimental and clinical studies [10–15]. This modality combines the benefits of conventional tidal expansions to maintain the lung open with high-frequency fluctuations facilitating axial gas mixing [16] and alveolar recruitment [12]. Despite improved gas exchange, superimposing the high-frequency component on conventional ventilation during inspiration results in higher peak pressures and tissues overdistension or lower tidal volumes in pressure control mode. Thus, the net benefit of this modality has been challenged [17, 18].

We hypothesize that using the high-frequency component during the expiration phase merely preserves the advantage of percussive ventilation without raising the peak inspiratory pressure and the combination of frequencies can reduce dynamic parenchymal strain and mechanical power [19–21], so preserving the lung from excessive stress. We contrasted this novel expiratory high-frequency percussive ventilation (eHFPV) with the whole-cycle HFPV and conventional mechanical ventilation to test this hypothesis. We aimed at assessing whether this eHFPV improved oxygen delivery and carbon dioxide (CO_2_) clearance in an experimental model of induced hypoxia and hypercapnia. Simulation research was also conducted to evaluate the effects of lung size on the oscillatory pressure transmission from the airway opening to the alveolar compartment in order to determine the extent to which our findings may be extended to other species.

## Methods

### Ethical considerations

This study was approved by the National Food Chain Safety and Animal Health Directorate of Csongrád County, Hungary (No. XXXII/149/2020) on March 10, 2020. The procedures were implemented in compliance with the guidelines of the Scientific Committee of Animal Experimentation of the Hungarian Academy of Sciences (updated Law and Regulations on Animal Protection: 40/2013. [II. 14.], the Government of Hungary), and European Union Directive 2010/63/EU on the protection of animals used for scientific purposes. The results were reported in line with the Animal Research Reporting of In Vivo Experiments (ARRIVE) guidelines.

### Animal preparation

Male New Zealand White rabbits (*n* = 10, weight: 2.0–2.5 kg) were sedated with an intramuscular injection of xylazine (5 mg/kg, CP-Xylazine; CP-Pharma, Burgdorf, Germany). An ear vein was cannulated with a 24-gage catheter (Abbocath, Abbott Medical, Baar/Zug, Switzerland) for drug delivery. Anesthesia was then induced and maintained through continuous intravenous infusions of propofol (10 mg/kg/h), fentanyl (5 µg/kg/h), and midazolam (0.2 mg/kg/h). Tracheotomy was performed under local anesthesia (subcutaneous injection of 0.5% lidocaine), and a 3.5-mm ID and 7-cm long uncuffed tube (Portex; Smiths Medical, Kent, UK) was inserted into the trachea. Rabbits were connected to a custom-made blower-driven ventilator and ventilated with room air (ventilation frequency, 20–25/min; tidal volume, 7 ml/kg; positive end-expiratory pressure [PEEP], 3 cmH_2_O; inspiratory-to-expiratory ratio, 1:2). A femoral artery and femoral vein were catheterized for drug delivery, blood sample collection, and blood pressure monitoring. After ensuring appropriate anesthesia depth, neuromuscular blockade was maintained via continuous infusion of atracurium (0.6 mg/kg/h, Tracrium; Aspen Pharma, Dublin, Ireland). The mean arterial pressure, heart rate, and electrocardiogram were monitored continuously during the experiment. A rectal probe was applied to monitor the body temperature, which was maintained at 38 °C–39 °C using a thermostatic heating pad (Harvard Apparatus, South Natick, MA, USA).

### Performing mechanical ventilation

**Fig. 1:**
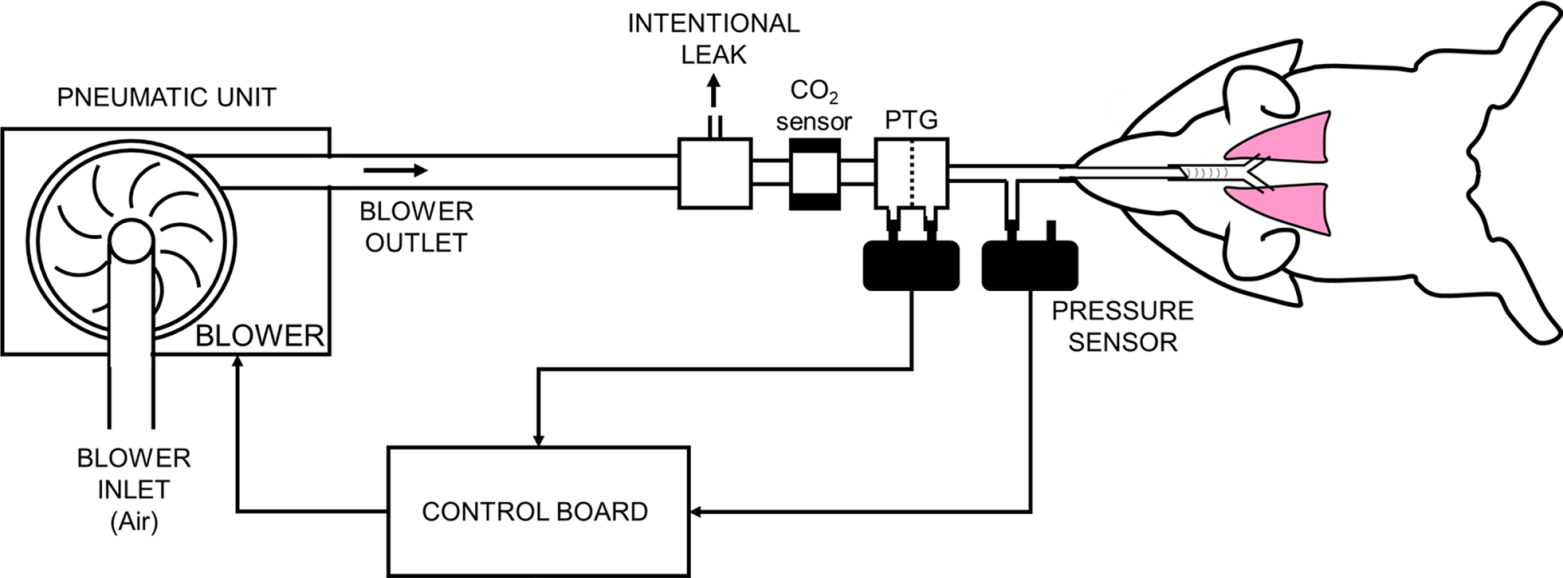
Scheme of the experimental setup. A custom-made blower-driven ventilator was used to deliver the driving pressure during conventional pressure-controlled ventilation, and it also allowed superposition of the high-frequency signals over the conventional pressure excursions. The rotation speed of the blower was controlled by a custom-made software to deliver the changes in the airway pressure required for the different mechanical ventilation modalities. Volumetric capnograms were recorded by using a mainstream CO_2_ sensor in series with a screen pneumotachograph (PTG)

The custom-made blower-driven ventilator was tailored to suit the delivery of the driving pressure in the conventional pressure control mode, as it allowed superposition of the high-frequency signals over the conventional pressure excursions in a controlled manner (Fig. 1). The required changes in the airway pressure pattern during the different mechanical ventilation modalities were generated by controlling the rotation speed of the blower with custom-made software. During conventional mechanical ventilation, the constant rotation speed of the blower was altered periodically to provide the required RR and VT. When HFPV or eHFPV were applied, the rotation speed of the blower was modulated by adding the frequency of the oscillatory component required in order to deliver these complex pressure waveforms.

### Blood gas analyses

For blood gas analyses, 0.1 ml of arterial blood samples were collected under each experimental condition. Partial pressures of oxygen (PaO_2_) and CO_2_ (PaCO_2_) along with oxygen saturation (SaO_2_) were measured by using a point-of-care blood analyzer system (Epoc Reader and Host; Epocal, Inc., Ottawa, ON, Canada).

### Volumetric capnography

Intra-tidal changes in the CO_2_ concentration were measured with a pediatric mainstream sensor connected to a data acquisition unit (Capnogard®; Novametrix, Andover, MA, USA). Central airflow was sensed by a screen pneumotachograph (11-mm ID, PNT3500; Hans Rudolph, Inc., Shawnee, KS, USA) that was connected to a differential pressure transducer (model 24PCEFA6D; Honeywell, Charlotte, NC, USA). Both the signals were digitized at a sampling rate of 256 Hz and then analyzed with a custom-made software. Volumetric capnogram curves were generated from the intra-tidal changes in CO_2_ concentration and the volume signals derived by integration of the airflow data were simultaneously recorded.

The inflection point of phase 2 was localized as the maximum of the first derivative of the volumetric capnogram curve. The maximum of the third derivatives of the capnogram before and after this inflection point were identified, and this range was considered as a capnogram phase 2 [22], which reflects mixed emptying of the airway-alveolar spaces. Phase 3 of the capnogram curves, which represents the expiration of the alveolar gas compartment, was defined as the range from the end of phase 2 until the end of expiration. Capnogram phase 2 slope (S2V) was derived by fitting a linear regression line to 5 points around the inflection point. Fowler’s anatomic dead space (VDF) was defined as the gas volume expired until the inflection point in phase 2 [23]. CO_2_ volume eliminated in a minute (V’CO_2_) was calculated as the product of expired CO_2_ within a breathing cycle (VCO_2_) and the ventilation frequency. VCO_2_ was obtained by integrating the expiratory phases of the volumetric capnogram curve within each ventilation cycle. Since the application of the HFPV and eHFPV caused substantial fluctuation in phase 3 of the capnogram (e.g., Fig. 3), the shape factors and dead space parameters related to this segment were significantly biased and not extracted accordingly.

### Ventilation monitoring

The pressure signal at the airway opening was recorded during the entire ventilation period. Tidal volume (VT), and minimum (P_min_), maximum (P_max_), and mean airway pressures (P_mean_) were derived from these signals under steady-state conditions.

### Measurement of respiratory mechanics

The forced oscillatory measurement of airway and respiratory tissue mechanics have been detailed previously [24]. Briefly, the custom-made blower-driven ventilator generated a small-amplitude (less than ± 1 cmH_2_O) pseudo-random signal containing 23 sinusoidal components ranging from 0.5 to 20.75 Hz during short apneic periods (8 s) that was interposed into the mechanical ventilation. Oscillatory airflow was measured with a screen pneumotachograph (11-mm ID, PNT3500; Hans Rudolph, Inc.) connected to a differential pressure transducer (model 24PCEFA6D; Honeywell), and tracheal pressure (Ptr) was sensed with an identical pressure transducer relative to the atmosphere. The input impedance spectra of the respiratory system (Zrs) were calculated as Zrs = P/V′. The input impedance of the tracheal tube and the connections also were measured with applying a reduced oscillatory amplitude in the open circuit that corresponds to the oscillatory amplitude employed in vivo, and the value obtained was subtracted from the Zrs spectra before further analyses.

The mechanical properties of the airways and respiratory tissues were determined by model fitting through minimizing of the weighted difference in the measured and modeled spectra. The well-validated model [25] contained a frequency-independent airway resistance (Raw) and inertance connected in series to a constant-phase tissue compartment incorporating damping (G) and elastance (H) parameters [26]. Since the frequency-independent resistive component of the chest wall is negligible in rabbits [27], the parameter Raw mainly represents the flow resistance of the conducting airways. Parameters G and H signify the resistive (i.e., damping or energy loss) and elastic (i.e., stiffness) characteristics of the respiratory tissues.

### Simulation study to model pressure transmission

A simulation study using a lumped-element model of the respiratory system was performed to estimate how the lung size affects the transmission ratio of the high-frequency oscillatory pressures from the airway opening (Pao) to the alveoli (Palv). The details and results of the simulation are provided in Additional file 1.

### Experimental protocol

**Fig. 2:**
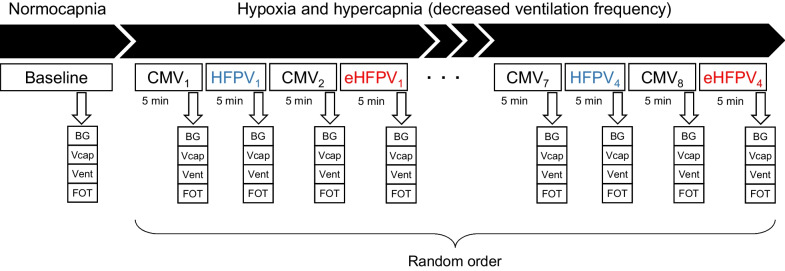
Experimental protocol scheme. Measurements were initially made under normocapnic conditions (baseline) and continued during the maintenance of hypoxia and hypercapnia by decreasing the ventilation frequency. Four sequences including a *high-frequency percussive ventilation* (HFPV) and an *expiratory high-frequency percussive ventilation* (eHFPV), each preceded by a *conventional mechanical ventilation* (CMV), was then performed. Both HFPV and eHFPV were applied by randomly setting the oscillatory amplitude (2 or 4 cmH_2_O) and the oscillatory frequency (5 or 10 Hz). BG: blood gas; Vcap: volumetric capnography; Vent: recording the mechanical ventilation pressure/volume parameters; FOT: forced oscillations

The scheme of the experimental protocol is demonstrated in Fig. 2. Hyperinflation was performed before initiating the protocol to standardize the volume history by maintaining an airway pressure of 25 cmH_2_O for 5 s. The animals were initially ventilated by conventional pressure-controlled ventilation (CMV). Normocapnia was maintained in phase 1 of the protocol by maintaining CMV, and the measurements were performed by analyzing blood gas, recording the volumetric capnogram and ventilation curves, and collecting forced oscillatory data epochs. To assess the ability of the novel ventilation strategy toward improving gas exchange, hypoxia and hypercapnia were induced by decreasing the ventilation frequency so as to attain a target PaCO_2_ of 53–56 mmHg during conventional ventilation. After establishing stable ventilation and hemodynamic conditions, another data set for blood gas, volumetric capnography, ventilation, and forced oscillations was collected while CMV was maintained (CMV_1_). Percussive ventilation modes were then initiated by randomly setting the oscillatory amplitude (2 or 4 cmH_2_O) or frequency (5 or 10 Hz) added either to the entire breathing cycle (HFPV) or to the expiratory phase alone (eHFPV). A 5-min period was allowed for the ventilation to reach a steady-state condition, and another set of data was collected under CMV and identical percussive ventilation to that of the earlier phase of the protocol. This randomized sequence was continued during the maintenance of hypercapnia until all percussive ventilation settings with amplitudes of 2 and 4 cmH_2_O and frequencies of 5 and 10 Hz were completed for both HFPV and eHFPV.

### Statistical analysis

The scatter of the values of the measured variables was presented as half-width of the 95% confidence interval. Blood gas, capnographic, and ventilation variables were compared by 2-way repeated measures analyses of variance with ventilation mode (CMV, HFPV, and eHFPV) and experimental stages (amplitude and frequency settings) as independent within-subject factors. Pairwise comparisons were performed via post hoc *t*-tests with Holm–Sidak corrections. Sample size was estimated to detect 10% difference in the primary outcome variable PaCO_2_ by using a power of 0.8 and 2-sided α error of 0.05. The estimation resulted in a desired sample size of 9 animals in each group, based on the blood gas data obtained in our earlier study under similar experimental conditions [28]. Statistical tests were performed within the *R* environment with the *lme4* [29] and *lsmeans* [30] program packages. p < 0.05 was considered to indicate statistical significance and all p values were 2-sided.

## Results

The baseline blood gas, capnographic, ventilation, and respiratory mechanical parameters are summarized in Table 1. Before inducing hypercapnia and hypoxia, all parameters were within normal limits.

**Table 1:** Mean ± half width of the 95% confidence interval of the blood gas, capnography, ventilation and forced oscillatory parameters obtained under the baseline condition when conventional mechanical ventilation was applied

Blood gas		Capnography		Ventilation		Forced oscillations	
PaCO_2_ (mmHg)	37.5 ± 3.4	VDF/VT (%)	37.5 ± 1.0	VT (ml)	24.6 ± 1.6	Raw (cmH_2_O.s/l)	12.8 ± 2.0
PaO_2_ (mmHg)	71.1 ± 7.6	S2V (mmHg/ml)	11.5 ± 0.6	P_max_ (cmH_2_O)	11.8 ± 0.8	G (cmH_2_O/l)	86.7 ± 16.6
SaO_2_ (%)	94.7 ± 2.1	V′CO_2_ (ml)	26.1 ± 2.2	P_min_ (cmH_2_O)	2.9 ± 0.01	H (cmH_2_O/l)	237.8 ± 22.0
				P_mean_ (cmH_2_O)	5.8 ± 0.1		

PaCO_2_: partial pressure of carbon dioxide in the arterial blood, PaO_2_: partial pressure of oxygen in the arterial blood, SaO_2_: arterial oxygen saturation. VDF/VT: dead space fraction according to Fowler, S2V: phase 2 slope of the volumetric capnogram, V’CO_2_: eliminated CO_2_ volume in a minute. VT: tidal volume, P_mean_: mean airway opening pressure, P_max_: peak airway opening pressure, P_min_: minimum airway opening pressure. Raw: airway resistance, G: respiratory tissue damping (viscous resistance), H: respiratory tissue elastance (stiffness)

**Fig. 3:**
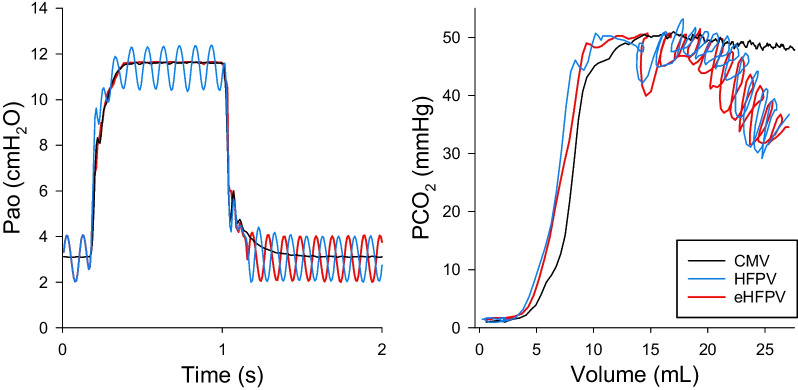
Left: Changes in the airway opening pressure during conventional mechanical ventilation (CMV, black), during *high-frequency percussive ventilation* (HFPV, blue) and an *expiratory high-frequency percussive ventilation* (eHFPV, red) (10 Hz oscillatory frequency administered with an amplitude of 2 cmH_2_O) obtained in a representative rabbit. Right*:* corresponding volumetric capnogram curve

Figure 3 depicts typical tracheal pressure signals during application of CMV, HFPV, and eHFPV in a rabbit by introducing 10 Hz pressure fluctuations with a pressure amplitude of 2 cmH_2_O applied. The reduction in VDF and S2V is shown by the corresponding representative volumetric capnogram curves.

**Fig. 4:**
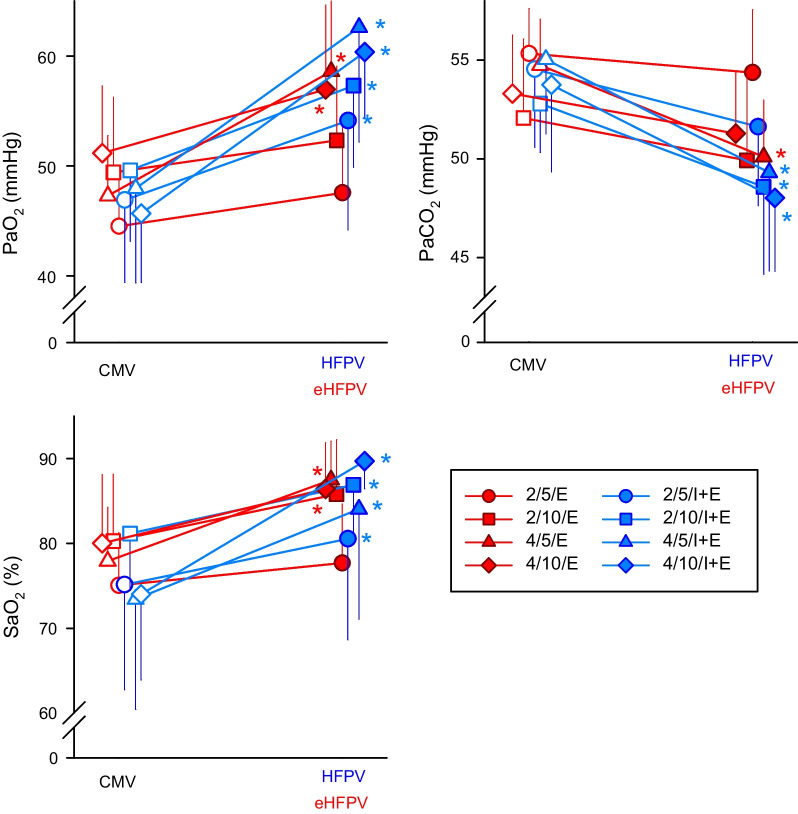
Arterial blood gas parameters during hypoxia and hypercapnia with normal mechanical ventilation (CMV, open symbols on the left), during *high-frequency percussive ventilation* (HFPV, blue filled symbols on the right) and during *expiratory high-frequency percussive ventilation* (eHFPV, red filled symbols on the right). Characters in the legend denote the oscillatory amplitude (2 or 4 cmH_2_O), the superposed frequency (5 or 10 Hz), and its application phase (E: expiratory phase only; IE: entire breathing cycle). PaO_2_: arterial partial pressure of oxygen, PaOC_2_: arterial partial pressure of carbon dioxide, SaO_2_: arterial oxygen saturation. *p < 0.05 between HFPV or eHFPV vs. the corresponding CMV

Figure 4 depicts the absolute values of the parameters acquired from arterial blood gas analyses during the different protocol stages, as well as their relative variations between the ventilation modes. The superposition of high-frequency oscillations throughout the whole ventilation cycle had a significant effect on the absolute values of PaCO_2_, PaO_2_, and SaO_2,_ regardless of the frequency or amplitude of the high-frequency component (p < 0.001 for all). Improvements in gas exchange were also observed when a high-frequency signal with an amplitude of 4 cmH_2_O and frequency of 5 Hz was applied in the expiratory phase only (p < 0.001 for all). While such improvement was evidenced in oxygenation indices (PaO_2_ and SaO_2_) at a frequency of 10 Hz (p < 0.01 for both), there was only a tendency for a significant increase in CO_2_ clearance at 10 Hz (p = 0.1).

**Fig. 5:**
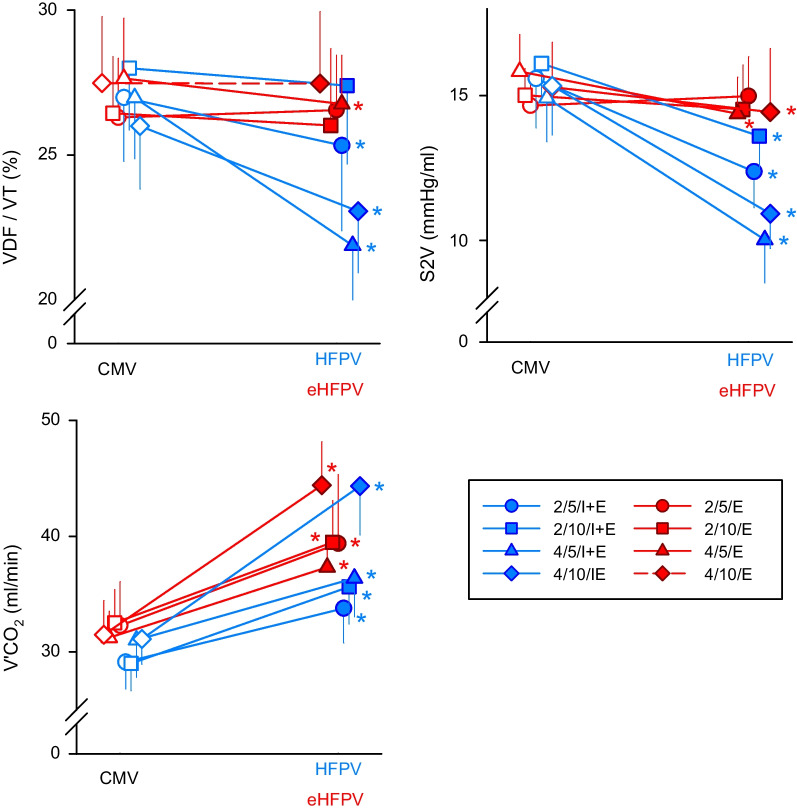
Parameters obtained via mainstream capnography during hypoxia and hypercapnia with normal mechanical ventilation (CMV, open symbols on the left), during *high-frequency percussive ventilation* (HFPV, blue filled symbols on the right) and during *expiratory high-frequency percussive ventilation* (eHFPV, red filled symbols on the right). Characters in the legend denote the oscillatory amplitude (2 or 4 cmH_2_O), the superposed frequency (5 or 10 Hz), and its application phase (E, expiratory phase only; IE, entire breathing cycle). VDF/VT: anatomical dead space fraction according to Fowler, S2V: phase 2 slope, V’CO_2_: minute elimination of carbon dioxide. *p < 0.05 between HFPV or eHFPV vs. the corresponding CMV

Figure 5 depicts the absolute values of ventilation parameters acquired by mainstream capnography and their relative variations between ventilation modes. HFPV improved capnography parameters (VDF/VT, S2V and V’CO_2_) independent of amplitude or frequency applied (p < 0.001 for all). While VCO_2_ elevated at all amplitudes and frequencies used during eHFPV (p < 0.001), this ventilation modality only improved VDF/VT and S2V at higher oscillatory amplitudes (p < 0.05 for all).

Additional file 1: Table S1 shows the relative differences in respiratory mechanical parameters between conventional and percussive ventilation modalities (Additional file 1). In comparison to CMV, neither HFPV nor eHFPV showed a significant effect on any airway or respiratory tissue parameters.

**Fig. 6:**
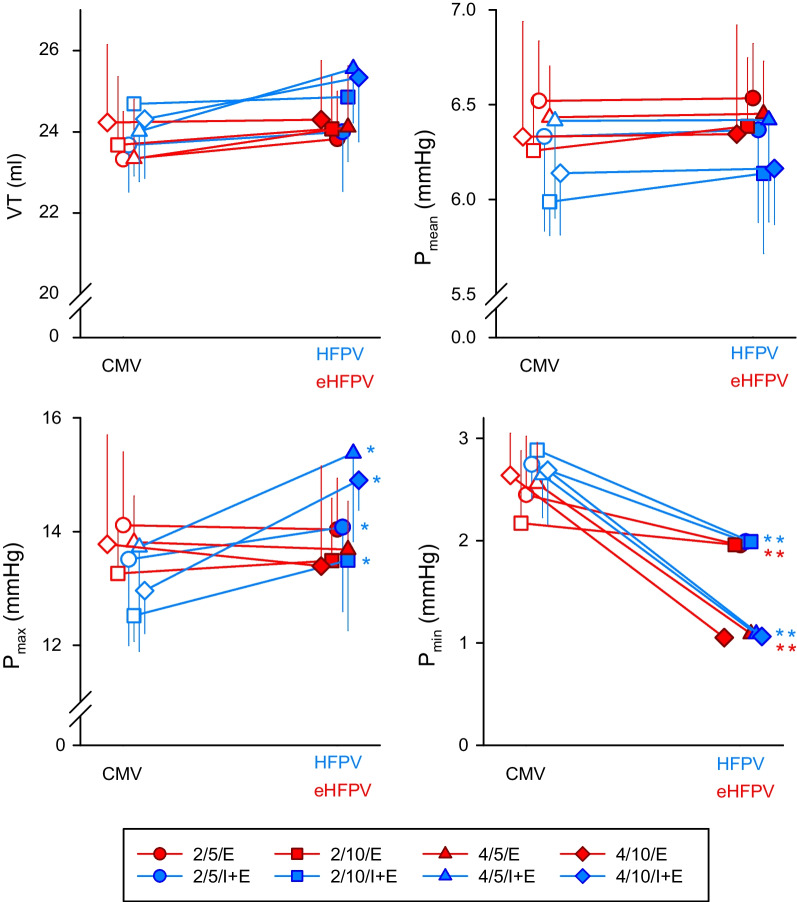
Tidal volume and ventilation pressures obtained during hypoxia and hypercapnia with normal mechanical ventilation (CMV, open symbols on the left), during *high-frequency percussive ventilation* (HFPV, blue filled symbols on the right) and during *expiratory high-frequency percussive ventilation* (eHFPV, red filled symbols on the right). Characters in the legend denote the oscillatory amplitude (2 or 4 cmH_2_O), the superposed frequency (5 or 10 Hz), and its application phase (E, expiratory phase only; IE, entire breathing cycle). P_mean_: mean airway opening pressure, P_max_: maximum airway opening pressure, P_min_: minimum airway opening pressure, VT: tidal volume. *p < 0.05 between HFPV or eHFPV vs. the corresponding CMV

Figure 6 summarizes the parameters representing the delivered VT and corresponding airway opening pressures for the various ventilation strategies. At any experimental stage, there was no difference in VT and P_mean_. The decreases in P_min_ during all forms of percussive ventilation mirrored the negative ramifications of high-frequency pressure fluctuations at the airway opening. P_max_ was only enhanced with HFPV because the oscillations in this modality persisted during the inspiratory phase.

Additional file 1 describe the relationship between lung size and the ratio of alveolar and airway opening pressures (Palv/Pao) derived from the simulations. Palv/Pao showed a rapid decline with FRC rising until around 400 ml, when it plateaued at roughly 45% and 25% for oscillation frequencies of 5 and 10 Hz, respectively.

## Discussion

The current study evaluated the usefulness of a novel ventilation approach based on the application of a high-frequency oscillatory component solely to the expiratory phase of invasive conventional single-frequency mechanical ventilation. The efficacy of this expiratory high-frequency percussive ventilation to increase O_2_ delivery and CO_2_ elimination with a 5 Hz oscillation frequency applied with a 4 cmH_2_O amplitude was demonstrated in rabbits with healthy lungs. These benefits in gas exchange were associated with decreased ventilation dead space and capnogram phase 2 slope, as well as enhanced CO_2_ minute elimination. This beneficial profile was not associated with alterations in the airway or respiratory tissue mechanics.

To satisfy the growing demand for improved ventilation assistance, many alternative types of mechanical ventilation have been developed. While PEEP elevation reduces pulmonary complications and improves patient outcomes [28, 31], this strategy is not without limits. High PEEP potentially increases stress and strain on lung tissues, and the reduced venous return and cardiac output, along with permissive hypercapnia [32], may result in cardiovascular impairment [33]. Another approach is application of variable tidal volume and frequencies based on prerecorded physiologic breathing patterns. The advantage of physiologically variable ventilation on respiratory mechanics, gas exchange, and lung inflammatory outcomes were established in healthy lungs [28], but not in severely injured lungs [33]. High-frequency oscillatory ventilation (HFOV) has been considered particularly in newborns but failed to demonstrate a benefit in adults particularly in the presence of ARDS characterized by a non-homogeneous insult, due to the risk for developments of distinct areas with cyclic hyper- and hypoinflation [1]. Alternatively, application of multi-frequency oscillatory ventilation in pediatric animal models of lung injury revealed improvements in oxygenation and CO_2_ elimination [20, 34] providing a clinical perspective to this ventilation modality as an advancement of HFOV.

CMV was coupled with superimposed constant-pressure fluctuations in our investigation, either throughout the whole ventilation cycle or during the expiratory phase. This approach combines the benefits of conventional tidal expansions to maintain the lung open with the high-frequency oscillations of HFOV. Previously, high oscillatory amplitudes were overlaid on conventional waveforms to apply HFPV [10–15]. Percussive ventilation with oscillatory amplitudes of 10–20 cmH_2_O improved gas exchange by enabling alveolar recruitment at the expense of greater peak inspiratory pressure [12]. With the use of modest amplitude oscillations, we were able to increase gas exchange in the current investigation (up to 5 to 10 times smaller than those applied previously during HFPV). As a result, our findings suggest that high frequencies delivered at significantly lower amplitudes are still effective at increasing gas exchange. While applying multi-frequency oscillatory ventilation with high frequencies (5–15 Hz) improved lung recruitment via a dithering effect to keep lung units open [34], the lack of improvements in respiratory mechanics (Additional file 1: Table S1) with HFPV and eHFPV waveforms with lower frequencies suggests that the enhanced gas exchange is not due to alveolar recruitment. Alternatively, the reduced VDF and S2V indicates augmentation of longitudinal gas transport via HFOV processes (Fig. 5), such as inducing asymmetric velocity profile, pendelluft, turbulence, molecular diffusion, and collateral ventilation [16]. Combining multiple frequencies in mechanical ventilation waveform may also improve gas exchange due to the highly frequency-dependent nature of how flow is heterogeneously distributed throughout the airway tree [19, 35–37]. Accordingly, these mechanisms have added value to the advantage of maintaining bulk flow and alveolar recruitment supplied by the CMV waveform component of HFPV and eHFPV.

The primary finding of the present study is the ability of eHFPV to improve gas exchange but when higher amplitude (4 cmH_2_O) and the lower oscillatory frequency (5 Hz) were superimposed on the conventional expiratory waveform. This oscillation amplitude was high enough to augment longitudinal gas transport, and the 5 Hz frequency was low enough to diminish the intrapulmonary shunting effect (Fig. 2S), resulting in better gas exchange. The improved axial gas mixing drained the alveolar CO_2_ gas content. In this context, the expired tidal volume can be considered virtually higher than the conventional inspiratory tidal volume. Likewise, oscillation is anticipated to restore the alveolar O_2_ fraction during expiration. During expiration, the intraluminal gas transfer can increase gas exchange without increasing inspiratory or expiratory pressures. Unlike in HFPV, this improvement during eHFPV did not result in an increase in peak inspiratory pressure (Fig. 6). High peak inspiratory pressure is the major cause of ventilator-induced lung injury (VILI) because it causes overdistension in the airway and alveolar compartments, resulting in the release of proinflammatory cytokines [38, 39]. Using oscillations solely during expiration to avoid excessive peak inspiratory pressure may protect against VILI, especially in lungs with restrictive diseases. This potentially protective feature of eHFPV takes on added significance in light of previous findings demonstrating that not only is increased alveolar stress and strain responsible for the inflammatory response in VILI, but excessive airway distensions also trigger proinflammatory bronchial responses, contributing to ventilator-induced lung injury [38].

The degree of improvement in gas exchange during percussive ventilation modes is determined by the efficiency with which the high-frequency component is delivered to the lung periphery [40]. High-frequency oscillations are attenuated by the resistive and elastic losses in the airways and gas-filled compartments, which are all dependent largely on lung size via complex mechanisms. Reduced shunting caused by narrower airspaces and less compliant tissue compartments in a smaller lung favors oscillatory signal transmission to the alveoli. Elevated resistive losses, on the other hand, reduce the oscillatory component of HFPV and eHFPV more significantly in smaller lungs. Our simulation study using representative values to characterize lung volumes and respiratory mechanics indicated that as lung size increased, so did the loss of oscillatory signal (Additional file 1: Fig. S2). As a result, more efficient transport of the high-frequency component to the alveolar spaces might be expected in smaller lungs (i.e., sizes in neonates and small children). This finding is consistent with the more evident benefit of HFOV in neonates and children without substantial ventilation heterogeneities, and also explains the controversy over the efficacy of HFOV in adults [1]. Subsequently, eHFPV may also be particularly beneficial in smaller lungs. However, our simulation study demonstrated that the oscillatory signal can be transmitted to the alveolar spaces of adult lungs with around 45% (5 Hz) and 25% (10 Hz) efficiency, indicating the presence of a gas exchange improvement even in adult-sized lungs.

The magnitude of changes in the gas exchange outcomes is noteworthy. While the improvements in PaCO_2_, PaO_2_, and SaO_2_ were statistically highly significant, the magnitude of changes was relatively modest (~ 10%–20%). Nevertheless, even small differences in these critical parameters can have a significant influence on the development of a gas exchange abnormality [2, 41]. Accordingly, prognosis for patient outcome may be fundamentally affected even by small favorable differences in clinical situations involving a progressive decline in gas exchange, such as in patients with acute lung injury and ARDS [41].

## Conclusions

In summary, our findings demonstrate the efficacy of HFPV in improving gas exchange even when its oscillatory component is markedly smaller in amplitude than applied conventionally. The reduced ventilation dead space, capnogram phase 2 slope, and enhanced minute elimination of CO_2_ were not associated with changes in respiratory mechanics, suggesting that augmented longitudinal gas transport was involved rather than an increased lung surface available for gas exchange. In an additional effort to reduce lung overdistension, a novel mechanical ventilation modality based only on the superposition of a high-frequency oscillatory component during the expiratory phase of the conventional waveform was developed. This modality, termed as expiratory high-frequency percussive ventilation (eHFPV), enhanced oxygen delivery and CO_2_ removal by carefully selecting oscillatory frequency and amplitude without increasing lung expansion. Our findings suggest that ventilation with eHFPV has the potential to reduce hypoxemia and hypercapnia without increasing the driving pressure at the airway opening. Confirmation of our findings in subsequent trials has the promise to facilitate modification of already existing HFPV ventilators to extend their abilities with the eHFPV modality.

## Supplementary Information

**Additional file 1:** Details of the simulation study and additional data for respiratory mechanics.

## Data Availability

The datasets used and/or analyzed during the current study are available from the corresponding author on reasonable request.

## Abbreviations

ARDS: Acute respiratory distress syndrome
CMV: Conventional mechanical ventilation
eHFPV: Expiratory high-frequency percussive ventilation
FRC: Functional residual capacity
G: Tissue damping
H: Tissue elastance
HFOV: High-frequency oscillatory ventilation
HFPV: High-frequency percussive ventilation
PaO_2_: Arterial partial pressures of oxygen
PaCO_2_: Arterial partial pressures of carbon dioxide
Palv: Alveolar pressure
Pao: Airway opening pressure
PEEP: Positive end-expiratory pressure
P_min_: Minimum airway pressure
P_max_: Maximum airway pressure
P_mean_: Mean airway pressures
Ptr: Tracheal pressure
Raw: Airway resistance
S2V: Capnogram phase 2 slope
SaO_2_: Arterial oxygen saturation
V’CO_2_: CO_2_ volume eliminated in a minute
VCO_2_: Expired CO_2_ within a breathing cycle
VDF: Fowler’s anatomic dead space
VILI: Ventilator-induced lung injury
VT: Tidal volume
