# Supplementary material for: Expiratory high-frequency percussive ventilation: a novel concept for improving gas exchange
Source: Respir Res. 2022 Oct 15;23:283. doi: 10.1186/s12931-022-02215-2 (PMC9569091; doi:10.1186/s12931-022-02215-2)
Supplement: Supplementary file 1 — Additional file 1: Details of the simulation study and additional data for respiratory mechanics. [file 12931_2022_2215_MOESM1_ESM.docx]

**Expiratory high-frequency percussive ventilation: a novel concept for improving gas exchange**

***Online data supplement***

Ferenc Peták^1^, Gergely H. Fodor^1^, Álmos Schranc^1^, Roberta Südy^1,3^, Ádám L. Balogh, PhD^2^, Barna Babik^3^, André Dos Santos Rocha^2^, Davide Bizzotto^4^, Raffaele L. Dellacà^4^,
Walid Habre^2, 5^

^1^ Department of Medical Physics and Informatics, University of Szeged, Szeged, Hungary

^2^ Unit for Anaesthesiological Investigations, Department of Acute Medicine, University of Geneva, Geneva, Switzerland

^3^ Department of Anaesthesiology and Intensive Therapy, University of Szeged, Szeged, Hungary

^4^ Dipartimento di Elettronica, Informazione e Bioingegneria, Politecnico di Milano, Milan, Italy

^5^ Paediatric Anaesthesia Unit, Department of Acute Medicine, University Hospitals of Geneva, Geneva, Switzerland

1. **Simulation study**

To estimate the transmission ratio of high-frequency oscillatory pressures from the airway opening (Pao) to the alveoli (Palv), we simulated pressure-flow relationships in the respiratory system by using a lumped-element model (Fig. S1). In this model, a resistor and an inductor represented the airway resistance (Raw) and the inertance (Iaw), respectively. The mechanical properties of the respiratory tissues were modeled by creating a parallel connection of a capacitor (Calv) representing compliance of the alveolar gas and a constant phase tissue compartment [1], incorporating dissipative (G) and elastic (H) elements. A capacitor, connected in parallel to the combination of the airway and respiratory tissue compartment (Caw), represented the airway compliance. The input parameters of the simulation were assembled from the present results and from previous studies [2-8], which reported Raw, G, and H in species with different lung sizes. To quantify Calv, alveolar gas volume was estimated as the difference in the functional residual capacity (FRC) and Fowler’s anatomic dead space (VDF). If the FRC or VDF were not reported in previousthe past studies; these parameters were calculated using reference equations [9, 10]. Simulations were performed for the oscillatory frequencies of 5 and 10 Hz.


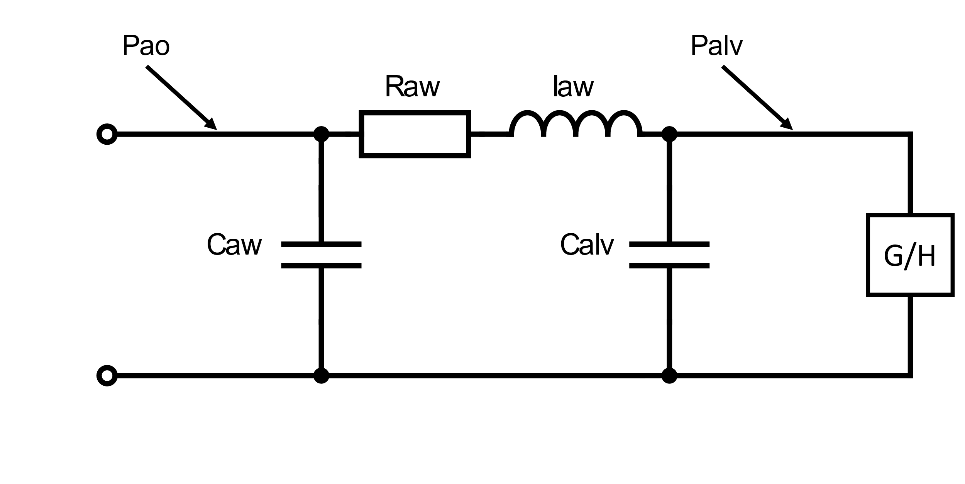


***Figure S1.*** *Schematic representation of the lumped-element model consisted of an airway and a tissue compartment. The airway compartment is represented by resistance (Raw), inertance (Iaw), and capacitance (Caw), while the tissue compartment is represented by the capacitance (Calv), and the constant phase damping (G) and elastance (H).*

Figure S2 depicts the relationship between FRC and the ratio of alveolar and airway opening pressures (Palv/Pao) obtained from the simulation study. Palv/Pao showed a steep decline with increasing FRC until reaching approximately 400 mL, which corresponded approximately to an end-expiratory lung volume of a 2-year-old child. A plateau phase appeared beyond this lung volume for both the oscillation frequencies, suggesting that approximately 45% of the 5 Hz and 25% of the 10 Hz oscillatory pressures applied at the airway opening reached the alveolar compartment at FRC values >400 mL.

***Figure S2.*** *The relationship among functional residual capacity (FRC), the ratio of alveolar (Palv), and airway opening (Pao) pressure was simulated using the lumped-element model based on previous studies on reporting airway and tissue mechanical parameters. In these studies, the results were obtained in rabbits (*● *[6]), children (*■ *[3], 🟊 [8] and* ▲*[5]), cynomolgus monkeys (*⬥ *[2]), teenagers (*⬢ *[4]), and adults (*▼*[7]). Simulations were performed at the oscillatory frequencies of 5 Hz (empty symbols) and 10 Hz (filled symbols), and the first-order hyperbolic regression curves were fitted to the simulated data (5 Hz, dashed line; R^2^ = 0.7; 10 Hz, continuous line; R^2^ = 0.76).*

1. **Supplemental data for the differences in respiratory mechanics**

|  | ***A/F/Phase*** | **Raw** (%) | **G** (%) | **H** (%) |
| --- | --- | --- | --- | --- |
| **HFPV** | **2/5/IE** | -1±4.71 | 2.31±5.62 | 0.08±3.01 |
|  | **2/10/IE** | 3.26±6.68 | 3.13±3.67 | -0.56±4.13 |
|  | **4/5/IE** | 2.52±6.52 | 3.36±2.81 | -1.66±3.78 |
|  | **4/10/IE** | 0.12±6.86 | 4.84±3.06 | -1.11±3.79 |
| **eHFPV** | **2/5/E** | 1.33±7.34 | 0.66±3.78 | 0.92±8.32 |
|  | **2/10/E** | -1.71±7.47 | 1.08±5.17 | 0.25±3.53 |
|  | **4/5/E** | 0.61±2.46 | -0.11±6.6 | -1.02±5.74 |
|  | **4/10/E** | -0.48±4.14 | 3.86±3.69 | 0.09±2.75 |

**Table S1.** Relative differences in the respiratory mechanical parameters among the conventional and the HFPV and eHFPV ventilation modalities with different amplitudes (first figure; 2 or 4 cmH_2_O), oscillation frequencies (second figure; 5 or 10 Hz), and application phases (third letters; E: expiration only, IE: inspiration and expiration).

REFERENCES

1. Hantos Z, Daroczy B, Suki B, Nagy S, Fredberg JJ: **Input impedance and peripheral inhomogeneity of dog lungs.** *J Appl Physiol (1985)* 1992, **72:**168-178.

2. Chapman RW, Skeans S, Lamca J, House A, Hey JA, Celly C: **Effect of histamine, albuterol and deep inspiration on airway and lung tissue mechanics in cynomolgus monkeys.** *Pulm Pharmacol Ther* 2005, **18:**243-249.

3. Petak F, Babik B, Asztalos T, Hall GL, Deak ZI, Sly PD, Hantos Z: **Airway and tissue mechanics in anesthetized paralyzed children.** *Pediatr Pulmonol* 2003, **35:**169-176.

4. Ionescu C, Desager K, De Keyser R: **Fractional order model parameters for the respiratory input impedance in healthy and in asthmatic children.** *Comput Methods Programs Biomed* 2011, **101:**315-323.

5. Hall GL, Hantos Z, Petak F, Wildhaber JH, Tiller K, Burton PR, Sly PD: **Airway and respiratory tissue mechanics in normal infants.** *Am J Respir Crit Care Med* 2000, **162:**1397-1402.

6. Habre W, Scalfaro P, Schutz N, Stucki P, Petak F: **Measuring end-expiratory lung volume and pulmonary mechanics to detect early lung function impairment in rabbits.** *Respir Physiol Neurobiol* 2006, **152:**72-82.

7. Babik B, Asztalos T, Petak F, Deak ZI, Hantos Z: **Changes in respiratory mechanics during cardiac surgery.** *Anesth Analg* 2003, **96:**1280-1287.

8. Von Ungern-Sternberg BS, Petak F, Hantos Z, Habre W: **Changes in Functional Residual Capacity and Lung Mechanics during Surgical Repair of Congenital Heart Diseases.** 2009, **110:**1348-1355.

9. Stocks J, Quanjer PH: **Reference values for residual volume, functional residual capacity and total lung capacity. ATS Workshop on Lung Volume Measurements. Official Statement of The European Respiratory Society.** *Eur Respir J* 1995, **8:**492-506.

10. Numa AH, Newth CJ: **Anatomic dead space in infants and children.** *J Appl Physiol (1985)* 1996, **80:**1485-1489.
